# Announcing the 2015 *Viruses* Young Investigator Prize and Graduate Student/Postdoctoral Fellow Travel Awards

**DOI:** 10.3390/v7020707

**Published:** 2015-02-12

**Authors:** Eric O. Freed

**Affiliations:** Head, Virus-Cell Interaction Section, Deputy Director, HIV Drug Resistance Program, Center for Cancer Research, National Cancer Institute, Building 535, Room 110, Frederick 21702-1201, MD, USA; E-Mail: efreed@mail.nih.gov; Tel.: +1-301-846-6223


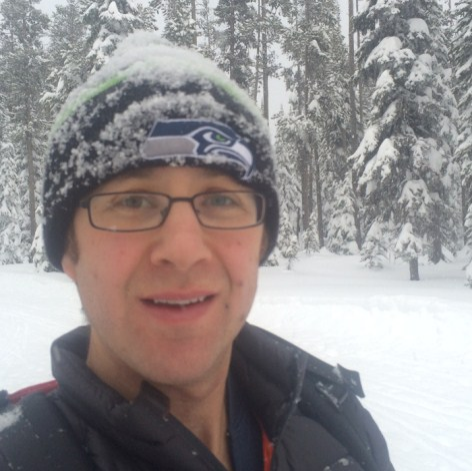

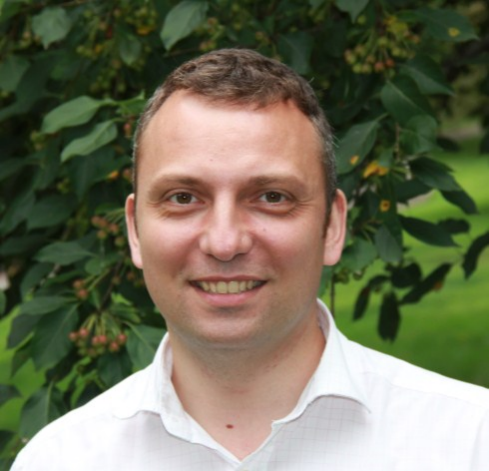
Winner: Dr. Jesse BloomRunner up: Dr. Alexander Ploss

With the goal of recognizing outstanding contributions to the field of virology by early-career investigators, last year *Viruses* accepted nominations for a 2015 Young Investigator Prize in Virology. The target age was set at 40 and under. Over 50 nominations were received and were evaluated by a panel of judges comprised of *Viruses* editorial board members.

I am pleased to announce Dr. Jesse Bloom of the Fred Hutchinson Cancer Research Center in Seattle, Washington, as the recipient of the 2015 *Viruses* Young Investigator Award.. Dr. Bloom received his Ph.D. with Dr. Frances Arnold and did postdoctoral training with Dr. David Baltimore, both at Caltech. Since 2011, he has been an Assistant Member in the Division of Basic Sciences at the Fred Hutchinson Cancer Research Center. Dr. Bloom’s research combines experimental and computational approaches to study influenza virus, with a focus on developing a better understanding of the biophysical and immunological constraints and selection pressures that shape influenza virus evolution. A major strength of his research is to seamlessly integrate molecular virology, next-generation sequencing, and computational biology to ask critical questions about influenza virus evolution. A highlight of Dr. Bloom’s postdoctoral research was an influential paper published in *Science* on the molecular basis for influenza virus resistance to oseltamivir (Tamiflu). More recently (*ELife* 2014), Dr. Bloom’s lab demonstrated that the “head” of the influenza hemagglutinin (HA) protein is highly tolerant of mutations. As the head of HA is the region of the protein that is targeted by antibodies, this finding sheds light on the remarkable ability of the influenza virus to escape the host immune response.

Dr. Bloom has received a number of awards and honors, including a Searle Scholar Award and a Sloan Research Fellowship.

When Dr. Bloom is not in the lab, he enjoys outdoor activities, such as running, hiking, and biking.

In addition to the cash prize and plaque, Dr. Bloom will be an invited speaker at the 2016 *Viruses* conference, Viruses 2016—At the Forefront of Virus-Host Interactions, to be held 26–28 January 2016, in Basel, Switzerland, http://sciforum.net/conference/viruses-2016.

Because of the outstanding quality of the nominations received for this award, we are also pleased to announce a runner-up: Dr. Alexander Ploss from Princeton University. Dr. Ploss conducted his Ph.D. research at the Memorial Sloan-Kettering Cancer Center in New York in the lab of Dr. Eric Palmer and did postdoctoral research with Dr. Charlie Rice at the Rockefeller University. In 2009, he was appointed Assistant Professor at the Rockefeller University and in 2013 he became an Assistant Professor at Princeton University. Dr. Ploss’s research is focused on addressing fundamental questions about hepatitis B and C virus infection using humanized mouse model systems. He is an author on over 60 scientific articles, many of which are published in high-impact journals. Dr. Ploss has received several honors and awards including the Astellas Young Investigator Award of the Infectious Disease Society of America and the Liver Scholar Award of the American Liver Foundation.

When not in the lab, Dr. Ploss is playing his violin, or, together with his wife, caring for their four young children.

In addition to the Young Investigator Award, *Viruses* sponsored a competition for travel awards for two graduate students or postdoctoral fellows to attend meetings of their choice. The 2015 travel award winners are Dr. Kirsty Short, a postdoctoral fellow in the lab of Dr. Thijs Kuiken, Department of Viroscience, Rotterdam, the Netherlands, and Dr. Redmond Smyth, a post-doctoral researcher in the lab of Dr. Roland Marquet, Institut de Biologie Moléculaire et Cellulaire, Strasbourg, France.

On behalf of the *Viruses* editorial staff and editorial board members, I wish to congratulate the four outstanding young virologists for their accomplishments.

